# Supplementary material for: Are carbon emissions trading and green financial instruments synergistic? -Comprehensive quantitative research based on content analysis
Source: PLoS One. 2024 Mar 7;19(3):e0298601. doi: 10.1371/journal.pone.0298601 (PMC10919601; doi:10.1371/journal.pone.0298601)
Supplement: S1 Data — (ZIP) [file pone.0298601.s001.zip › Data/地级市Z_F工作报告.zip!/╝¬┴╓/╦╔╘¡╩╨/└┤╫╘╣1⁄2╓┌║┼í╛┬φ┐╦╩2╛▌═°í┐_2016.docx]

各位代表：

　　现在，我代表市人民政府，向大会作工作报告，请各位代表连同《松原市国民经济和社会发展第十三个五年规划纲要（草案）》（以下简称《纲要》）一并审议，并请市政协各位委员提出意见。

　　一、“十二五”时期国民经济和社会发展回顾

　　 “十二五”期间，面对经济发展新常态，面对原油量价齐跌的巨大压力，面对抗洪抢险、抗震救灾的严峻挑战，面对革弊鼎新、正风肃纪的政治考验，我们在省委、省政府和市委的正确领导下，团结带领全市各族人民，深入贯彻习近平总书记系列重要讲话精神，认真落实“四个全面”和“三个五”战略部署，坚持稳中求进工作总基调，积极适应新常态，科学施策、克难前行，较好地完成了各项目标任务，各项事业都取得了重要成就。

　　综合经济实力实现新提升。主要经济指标实现稳健增长，经济总量在全省第三的位次上继续壮大。初步核算（下同），2015年全市地区生产总值实现1680亿元，同比（下同）增长6.3%；全社会固定资产投资完成1310亿元，增长12.2%；社会消费品零售总额实现610亿元，增长9.6%；外贸进出口总额达到1.4亿美元，增长19.5%，增速居全省第一位；城镇和农村常住居民人均可支配收入分别达到28245元和9571元，分别增长3.5%和10%；地方级财政收入完成50.2亿元。全市地区生产总值、社会消费品零售总额、外贸进出口总额分别是2010年的1.5倍、1.8倍和1.8倍。“十二五”期间，地方级财政收入累计完成276亿元，全社会固定资产投资累计完成5322亿元，累计引进域外资金3823亿元，分别是“十一五”时期的2.3倍、1.7倍和2.5倍。在经济稳步发展的同时，成功跻身“中国最具幸福感百强城市”，荣获全国文明城提名城市和省级双拥模范城称号，被国务院列为全国唯一的生物质能源产业集群城市和全省唯一的成长型资源城市，松原的知名度和影响力与日俱增。

　　经济转型升级实现新突破。积极培育工业竞争新优势。经开区晋升为国家级开发区，石化园区获批建立。扎实开展招商引资和项目建设攻坚活动，350个投资亿元以上工业项目建成投产，乾源50万立方米LNG、盼盼食品工业园、中化长山改扩建、苏源50万吨复合肥、新研牧神农机、阳光汽车部件、尼普洛嘉恒医药包装等一批大项目投产达效。吉林油田公司油气产量当量达到3282万吨。2015年规模以上工业增加值达到657亿元，是2010年的1.3倍。油气开采化工、农产品加工、装备制造、化肥等多元化产业发展格局初步形成。着力激活服务业发展潜力，服务业增加值达到650亿元。欧亚购物中心、新玛特购物广场等高端商贸体建成运营，中心城区商圈建设初具规模。查干湖和哈达山综合开发全面推进，沿江水利风景区和哈达山景区均晋升为国家级水利风景区，市规划展览馆晋升为4A级旅游景区。中东帕萨迪纳、亚泰澜熙郡等高端房地产项目相继建成。引进域外银行业金融机构5家，新增证券、保险、担保和小额贷款公司88家。全市银行业金融机构本外币各项存款余额达到942亿元、累计发放各项贷款1848亿元。市城开公司发行企业债券24亿元。“十二五”期末，民营经济增加值实现789亿元，是“十一五”期末的1.6倍。

　　 “三农”工作实现新转变。2015年一产增加值实现285亿元，是2010年的1.5倍。松原农业高新区获批国家农业科技园区，哈达山生态农业旅游示范区跨入省级开发区行列。市农科院组建成功获批。哈达山水利枢纽工程基本完工，主要设计功能全面发挥。松原灌区、河湖连通、引松入扶等重大农业基础设施项目建设稳步推进。前郭灌区和华侨农场建成省级高标准农田示范基地。开发整理土地165万亩。实施膜下滴灌255.6万亩。粮食总产量达到750万吨阶段性水平，我市连续三年被评为全国粮食生产先进市。前郭县被省政府评为“率先实现粮食生产全程机械化示范县”。成功组建松粮集团。市级以上农业产业化重点龙头企业和新型农民专业合作社分别新增45户和2000个。农村土地流转面积新增100万亩。有机、绿色、无公害农产品认证总量达到159个。新建标准化规模养殖场（小区）888个，中粮健康生猪产业化二期、正邦200万头生猪养殖加工一体化一期、齐全100万头生猪养殖加工一期等项目投产达效，长岭县被确定为全国“粮改饲”试点县。新农村建设扎实推进，建设新型农村示范社区300个，完成“四清”、“四改”村713个，新建农村公路1600公里，有效解决58万农村人口饮水安全问题。

　　经济社会焕发新活力。新一轮政府机构改革圆满完成，在全省率先实现非行政许可“零审批”，政府行政职权和行政审批事项分别减少1417项和150项，简政放权工作实现历史性突破。完成扶余撤县设市。出入境检验检疫机构成功组建。事业单位分类改革稳步推进。不动产登记完成职责整合、机构组建并实现登记发证。商事制度改革不断深化，全面实施“五证合一、一照一码”。财政预决算公开和国库集中支付制度改革深入推进，“营改增”稳步实施。前郭农信社成功改制为农商行。农村土地确权登记颁证、农村集体资产产权、“粮食银行”、扩权强镇等改革试点扎实推进。集体林权制度改革基本完成。中小学教师职称制度改革试点经验在全国推广。县级公立医院改革和基层医疗卫生机构基本药物制度全面实施。市县两级文化广电新闻出版局、广播电视台均完成整合组建，国有文艺院团成功改制。

　　城市建设展现新风貌。编制完成《松原市城市总体规划》和城市风貌、城市公共交通、地下综合管廊等20个专项规划。中心城区建成区面积达到56平方公里，人口达到49.6万。城镇化率达到43%，比2010年提高4.4个百分点。“美丽家园”行动计划顺利实施，累计投资511亿元，建设重大城市基础设施项目60个。一江两岸综合开发扎实推进，生态环保型壅水坝投入使用，天河大桥桩基工程基本完工。完成乌兰大街、青年大街、长宁街、松原大路、郭尔罗斯大路等63条街路新建、续建或改造任务，改造背街巷路90条。改造排雨排污管网41.2公里，雨污分流率提高20%。新建成吉思汗、纳仁汗等公园8处，高标准完成中山大街、五色广场等85处街路、广场绿化任务，城市人均公共绿地面积达到17.8平方米。松花江大桥桥体和东镇大路、滨江大道等街路及周边楼体亮化全面升级。松原内陆港建成运营，哈大客专松原段、松陶铁路、大广高速松原段投入使用，国道203线和省道304线、301线完成升级改造，松原成为全省首个拥有绕城高速的地级城市；查干湖机场、长白铁路扩能改造、松原客运交通枢纽与站前综合改造、宁江客运站等重大项目建设进展顺利，立体化大交通格局初步形成。

　　幸福松原建设迈上新台阶。连续组织实施民生行动计划，累计办民生实事322件，一大批涉及群众切身利益的热点难点问题得到解决。新增城镇就业24万人，安置再就业14.5万人，城镇登记失业率稳控在4%以内。转移农村劳动力270万人次。养老保险制度实现城乡全覆盖，建立12349养老信息平台，政府购买居家养老服务试点启动实施。医疗保险实现省内异地就医即时结算。失业、工伤、生育三项保险不断扩面提标。发放城乡低保资金4.4亿元、各类救助资金4100万元。市社会福利服务中心和救助站建成使用。市残疾人康复中心和托养中心全部竣工。改造城市棚户区166万平方米、农村危房3万户，建设安置住房2.2万套、廉租房22.4万平方米。完成“暖房子”工程350万平方米。综合整治老旧小区和弃管楼360万平方米。发放住房公积金贷款22.9亿元。新建、改造供热管网280公里，实现前郭县城和市城区统一供暖。江南新水厂建成投入使用，新建大型二次供水站24座，撤并老旧小型二次供水站112座，城区居民安全供水实现全覆盖。铺设天然气管网125公里，新增管道用户12万户，城区管道气化率提高55个百分点。新一代天气雷达系统建成使用。市城区地下活断层探测与地震危险性评价项目全面完成。高效有序应对严重地震灾害，创造了“无一人重伤、无一人死亡”的抗震救灾奇迹。投入资金9亿元，维修、新建房屋22680套，建设公共设施13.7万平方米，地震灾区建成崭新家园。

　　社会事业发展取得新成就。顺利实施科技项目150个，获省级科技进步奖25项、授权专利658件。新建、改扩建中小学校舍55万平方米、幼儿园406所，改造薄弱学校230所，建成示范性中小学和优秀村级小学各100所，市特殊教育学校完成移址新建。市中西医结合医院被评为三级甲等医院，市中心医院异地新建项目开工建设，全市1073所村卫生室全面完成标准化建设。市文化“三馆”投入使用，新建乡镇综合文化站78个、文化小广场572个，农家书屋和农村文化大院实现行政村全覆盖。满族新城戏《洪皓》获全国精神文明建设“五个一工程”奖，马头琴制作技艺、博舞等4个项目列入国家级非物质文化遗产名录，广播和电视人口覆盖率均达到98%。圆满承办查干湖全国公路自行车冠军赛等国省级体育赛事。查干湖冬捕节越办越好，影响越来越大。我市残疾人运动员成为吉林省历史上首位残奥会金牌获得者并创世界纪录。松花江流域水污染防治规划项目和“十二五”减排目标全面完成，“三化”草原治理、湿地修复工程取得明显成效。

　　民主法治建设获得新进展。自觉接受市人大及其常委会法律监督、工作监督和市政协民主监督，办理市人大代表建议308件、市政协提案652件，面复率、办复率和满意率逐年提高。法治松原建设和“六五”普法成效明显，市信访法律服务中心完成设立。依法行政、行政复议和行政执法监督工作进一步强化，审计监督和行政监察力度不断加大。坚决贯彻中央“八项规定”，扎实开展党的群众路线教育实践活动和“三严三实”专题教育，严肃整治“四风”问题，行政效能大幅提升。严格落实信访包保稳控责任，累计化解信访积案950件。安全生产和食品药品安全监管力度加大，各类突发事件得到及时妥善处置。社会治安防控体系更加完善，命案侦破、打黑除恶、“清网行动”和“打盗骗、保民安”战役成果显著。此外，双拥共建、统计、档案、质监、民族、宗教、外事、侨务、水文、人防、地方志、红十字等工作都取得了新成绩。

　　各位代表！过去五年，是我市发展进程中极不平凡的五年，发展困难超出预期，发展成就来之不易。今天的松原，已经站在基础更坚实、前景更广阔的全新起点上。这些成绩的取得，是省委、省政府和市委统揽全局、正确领导的结果；是市人大和市政协有效监督、大力支持的结果；是在历届政府和老领导、老同志打下的坚实基础上，全市广大干部群众团结拼搏、锐意进取的结果；是中省直单位、驻松部队、武警官兵、消防战士和社会各界热情参与、携手共建的结果。在此，我代表市人民政府，向所有为松原发展建设作出贡献的同志们、朋友们，致以崇高的敬意和衷心的感谢！

　　五年实践，我们深刻地感受到：只要坚决贯彻落实党中央、国务院和省委、省政府战略决策部署，坚持加快发展，抢抓机遇、乘势而上，就一定能够在振兴发展的道路上阔步向前；只要牢固树立科学发展理念，坚持科学决策，审时度势、务实重行，就一定能够创造出无愧于人民、经得起历史检验的业绩；只要不断推动体制机制创新，坚持深化改革，激发潜力、释放活力，就一定能够赢得广阔的发展空间；只要紧紧围绕建设幸福松原，坚持以人为本，依靠人民、服务人民，就一定能够凝聚起更加强大的发展合力；只要持之以恒地开拓进取，坚持脚踏实地，持续接力、连续发力，就一定能够写好松原发展的新篇章！

　　各位代表！在看到成绩的同时，我们也清醒地认识到，相对落后仍然是松原最基本的市情，从转型发展到全面建成小康社会还面临不少困难和问题：“一油独大”的产业格局还没有根本改变，产业结构不合理，地方工业发展滞后；内生动力不足，持续发展乏力，新的增长点不多；对外开放度不高，与经济总量在全省第三的位次还很不相称；现代农业科技水平不高，农业基础条件和资源优势尚未有效转化为经济优势；城乡基础设施建设欠账较多，城镇化步伐缓慢；经济、金融、信息等方面高层次人才短缺；脱贫攻坚任务艰巨，保障和改善民生压力加大；生态修复和环境保护形势较为严峻，大气和水污染还需深度治理；行政效能有待提升，经济发展软环境需要进一步优化，等等。对此，我们一定高度重视，在今后工作中认真加以解决。

　　二、《松原市国民经济和社会发展第十三个五年规划纲要（草案）》说明

　　 “十三五”时期，是松原贯彻落实中央“四个全面”战略布局和“创新、协调、绿色、开放、共享”发展理念以及省委“五个优势、五项举措、五大发展”战略的关键期，是加快推进转型发展的攻坚期，是全面建成小康社会的决胜期。《纲要》准确把握国家和省一系列方针政策，突出松原市情和发展阶段性特征，提出了未来五年我市经济社会发展的指导思想、奋斗目标和主要任务。下面，我作简要说明。

　　（一）“十三五”时期经济社会发展的总体思路和主要目标

　　按照市委要求部署，未来五年松原经济社会要实现又好又快发展，必须全面贯彻党的十八届五中全会、省委十届六次全会和市委五届六次全会精神，深入贯彻习近平总书记系列重要讲话精神，紧紧围绕中央“四个全面”战略布局和省委“三个五”战略部署，牢牢抓住新一轮振兴东北重大机遇，全面落实西部生态经济区建设战略，加快建设绿色产业城市和生态宜居城市，全力打好稳增长保卫战、调结构攻坚战、促发展持久战，协同推进新型工业化、城镇化、信息化和农业现代化，确保如期全面建成小康社会。

　　按照这个总体要求，未来五年我市经济社会发展的主要预期目标是：地区生产总值年均增长7%左右，地方级财政收入与经济保持同步增长，全社会固定资产投资年均增长12%，三次产业比重调整到14 ∶ 44 ∶ 42，城镇化率达到52%，单位地区生产总值能耗、二氧化碳排放强度、主要污染物排放总量持续减少，城乡常住居民人均可支配收入与经济增长同步，现行标准下贫困人口全部脱贫。

　　各位代表！“十三五”时期目标的确定，重点考虑了以下因素。一是适应经济发展新常态。当前，我市经济结构正处于增速换挡、结构优化、动力转换的重要时期，不能以浪费资源、牺牲环境为代价，片面追求经济增长，必须摆脱“速度依赖症”，推动发展方式加快从规模速度型向质量效益型转变。同时，又要保持经济适度增长，为转型发展创造良好条件。二是契合松原发展阶段特征。我市经济转型需要经历艰难复杂的过程，特别是原油价格低位徘徊、粮食价格走低的状况短期内难有明显改变，现有支柱产业发展受到严重制约，而医药健康、生物技术、新能源等战略性新兴产业从培育到形成规模需要一个累积的过程，经济增长动力转换还处在“进行时”。三是把握面临的重大机遇。国家“一带一路”建设的实施，为我市开放发展提供广阔空间；新一轮振兴东北战略的实施，为我市转型发展提供重大支撑；西部生态经济区战略的实施，为我市绿色发展提供强大动能；吉林省率先实现农业现代化的目标，为我市统筹发展提供重要契机；“互联网+”行动计划的实施和全面推进深化改革，为我市创新发展提供强劲动力；国家和省在松花江流域综合整治、棚户区改造、城乡危房改造和城市地下综合管廊建设等领域投资力度加大，机场、铁路、高速公路等重大基础设施建设工程迎来新一轮投资热潮，为我市共享发展提供有利条件。我们要主动适应新常态，科学把握新常态，积极引领新常态，始终保持建设绿色产业城市和生态宜居城市的战略定力，振奋精神、抢抓机遇，团结奋斗、真抓实干，努力开创“十三五”时期经济社会发展新局面！

　　（二）“十三五”时期的主要任务

　　《纲要》围绕打造绿色产业城市和生态宜居城市，确定未来五年发展的主要任务是：经济发展方面，紧紧围绕油气开采化工、农产品加工、特色旅游和商贸物流四大主导产业，全力打造“135”产业体系：打造油气开采化工和精细化工1个千亿级的产业集群，打造生物质资源综合利用、食品加工、生物医药和健康养生3个五百亿级的产业集群，打造特色旅游、商贸物流、化肥、装备制造、信息服务和电子商务5个百亿级的产业集群。同时，全力壮大开发区、县域和民营经济板块，加快做强三大经济增长极。城镇建设方面，加强对城市的空间立体性、平面协调性、风貌完整性、文脉延续性等方面的规划和管控，着力构建松原市区、扶余市区一主一副两个城镇发展核心区，以长白铁路、珲乌高速和哈大铁路、哈长高速为城镇发展轴带，以长岭、乾安两个县城为次级发展中心，以长山、三井子等15个重点镇为节点，打造“两核两带、两极多点”的城镇化发展格局。全面推进城镇基础设施建设和中心城区江南江北均衡发展，力争到2020年基本完成现有城镇棚户区、城中村和危房改造。扶贫开发方面，按照习近平总书记“六个精准”要求，坚持“实事求是、因地制宜、分类指导、精准扶贫”方针，攻坚拔寨，全力冲刺，确保2018年实现农村贫困人口全部脱贫。改善民生方面，积极顺应全市人民过上更好生活的新期待，围绕人民群众的生存需求、发展需求和安全需求，全面发展科技、教育、医疗、文化、体育等各项社会事业，推进公共安全建设，大力组织实施民生行动计划，让全市人民得到更多实惠。深化改革方面，按照党中央、国务院和省委、省政府的顶层设计，全面深化国有企业改革，扎实推进农村综合改革，加快金融改革创新，深化行政审批制度改革，最大限度释放发展活力。扩大开放方面，积极适应经济全球化新形势，牢牢把握国家“一带一路”、长吉图开发开放等战略，提高我市对外经贸的规模和水平，加快形成外向型产业集群，逐步构建全方位、宽领域、多层次的开放格局。

　　要实现上述目标任务，我们必须摆脱“路径依赖”和体制机制束缚，加快转变发展理念。一是突出创新驱动。要把创新摆在振兴发展的突出位置，坚持变中求新、变中求进、变中突破，全面推进体制创新、科技创新、管理创新、模式创新和业态创新，培育创新主体，扶持创新技术，引进创新企业，强化创新支撑，做好无中生有、有中生新的文章，让创新驱动成为推动松原经济滚石上山、爬坡过坎的强劲引擎。二是突出绿色带动。科学布局生产空间、生活空间和生态空间，用绿色生态技术改造三次产业，深入开展大气、土壤和水污染专项治理，加快形成绿色发展方式和生活方式，让生态环境成为改善人民生活质量的增长点。三是突出资本撬动。全面盘活存量资源资产，特别是国有耕地、林地、草场、水面、湿地以及闲置土地和厂房等资源，活化存量资本，嫁接增量资产，推动资源变资产、资产变资本、资本变资金，促进产业转化升级，充分发挥比较优势，积极构筑竞争优势，让资本撬动成为松原转型发展的助推器。

　　三、2016年主要工作

　　各位代表！2016年是全面建成小康社会决胜阶段的开局之年，是松原“经济增速筑底企稳、经济结构深度调整、经济动力接续转换”的关键一年。我们面临的压力巨大，但拥有的潜力更大。要进一步强化战略意识和机遇意识，牢固树立和贯彻落实中央“五大发展理念”，主动适应新常态，科学把握新常态，积极引领新常态，坚持稳中求进工作总基调，坚持稳增长、调结构、惠民生、防风险，着力加强供给侧改革，认真落实市委抓住“一个统领”、建设“两个城市”、打好“三大战役”的战略部署，鼓足干劲、迎难而上，开拓进取、真抓实干，确保实现“开门红”。

　　综合考虑各方面因素，2016年全市经济社会发展的主要预期目标是：地区生产总值实现1800亿元，增长6.5%以上；地方级财政收入增长3%；全社会固定资产投资完成1480亿元以上，增长13%；社会消费品零售总额完成665亿元，增长10%；城乡常住居民人均可支配收入与经济增长同步。完成上述目标，要重点做好以下工作：

　　（一）强化油气产业支撑。坚持“油地一家”理念，同舟共济、携手并进，促进地企深度合作。大力推进油气勘探。支持吉林油田公司加快常规油、致密油和深层致密气勘探，新增探明原油地质储量1000万吨。全力稳定油气生产。支持吉林油田和东北油气两家公司稳油增气，扎实推进龙凤山区块开发，开工建设中石化长岭201井试采厂，争取油气产量当量达到507万吨。稳步推动油气资源转化。盘活四氢呋喃装置，大力推进众诚10万吨丙烯、河南天伦10万立方米LNG等项目建设，突出抓好众诚页岩油原位裂解中试基地项目。积极助力油田减负发展。建立常态化沟通协调机制，综合开发利用吉林油田闲置资源，加快推进“四供一业”移交和油企“退城进园”。

　　（二）强力发展地方工业。坚持多元化发展方向，加快推进产业结构调整，不断增强工业经济实力。突出壮大主导产业。积极发展精细化工产业，规划建设精细化工产业园，开工建设辽宁瑞兴液体油深加工项目，加快推进200万吨/年碳基新能源化工项目落地。着力发展农产品精深加工产业，积极推动嘉吉生化90万吨扩能等项目，开工建设110万吨玉米深加工、50万吨绿豆全籽粒高值化综合利用等项目，加快推进鲁花花生油生产和100万吨稻米精加工、10万吨工厂化生产食用菌等项目。突出发展优势产业。加速发展医药产业，规划建设医药健康产业园，促进瑞思扬医疗器械及保健酒生产项目建成投产，全力支持百年汉克等医药企业做大做强，大力引进关联企业落户松原。着力发展装备制造产业，积极促进油田企业配套服务本地化，推动吉林石油装备技术工程服务公司开拓中东、独联体等域外市场，谋划建设电动汽车产业园，完成新研牧神农机扩大生产和配套建设，开工建设沈铁三江港电缆生产、天德玻璃钢管道等项目。推动史丹利60万吨复合肥等续建项目竣工投产，开工建设中化长山15万吨尿基复合肥和10万吨缓控释肥、云天化20万吨钾肥等项目。突出培育新兴产业。扶持发展生物技术产业。规划建设生物科技产业园，开工建设本然生物500万吨土壤调节剂项目，加快推动酵王生物酵之谷、三零生物产业园、味之源调味品产业园和上海华新生物制剂项目落地，全力引进上下游企业，促进产业集聚集群发展。积极推进全国生物质能源示范市建设，规划建设生物质能源产业园。充分发挥秸秆收储公司作用，综合收储秸秆不低于200万吨。推动20万吨生物基材料产业园项目投入生产，加快推进中化5万吨纤维素乙醇、富德生物质能源项目落地。扎实推进百公里输水干渠太阳能光伏发电及资源综合利用项目。深化与中国兵器工业新技术推广研究所和中海油集团等方面的战略合作，加快推进军民融合科技产业园、中海油工业烟气综合治理、先锋科技直饮水和一江两岸地热综合利用项目落地。

　　（三）加快发展现代农业。积极构建现代农业产业体系、生产体系和经营体系，争做全省农业现代化领头羊，正常年景全市粮食总产量达到750万吨。紧扣绿色农业。规划建设国家级三江口现代农业示范区，修复华侨农场5.1万亩耕地土壤，突出抓好前郭灌区绿色水稻、长岭绿色谷子、扶余四粒红花生、乾安绿色葡萄、宁江绿色有机蔬菜等8个示范基地建设，“三品”种植面积达到190万亩。紧扣规模农业。加快农村土地流转服务中心和交易平台建设，支持有实力的企业投资发展现代农业，鼓励农村能人、种粮大户组建专业合作社和家庭农场，新增新型农业经营主体1030个，土地规模经营面积达到375万亩。紧扣设施农业。完成哈达山水利枢纽附属工程建设收尾，加快推进松原灌区、河湖连通、引松入扶等项目，启动三江口灌区工程前期工作，新打机电井2865眼，农田旱涝保收面积达到耕地总面积的31.2%。新增农机总动力20万千瓦、农机作业量1000万标亩。紧扣高效农业。组建市农科院。推广高效立体种养和设施栽培技术，大力发展瓜果、食用菌等特色种植，建设特色农业种植基地。开展作物工厂化种植试点。建设粮油高产示范区95万亩。组建棚膜经济投资公司，棚菜种植面积发展到10万亩。紧扣绿色牧业。启动建设无抗养殖示范市，开展养殖场污染物综合处理和资源化利用，新建标准化规模养殖场（小区）170个，加快推进特沃斯万头精品肉牛养殖屠宰加工续建项目，开工建设乾羊牧业百万只肉羊产业化二期、大北农40万头生猪养殖屠宰加工等项目，推动吉林特驱100万头生猪养殖一期项目投入运营。紧扣智慧农业。加强农业物联网建设与应用。依托大型电商和市农网等平台，创建绿色农产品旗舰店，及时发布“三品”生产企业信息，提高农产品高端市场占有率。紧扣绿色中药材。制定支持中药材产业发展政策，积极推进特色中草药种植，建设规范化中草药和种苗种植基地2个，推广种植面积不低于3500亩。

　　（四）全力做大现代服务业。坚持激活存量、培育增量，推动各类业态统筹协调发展，有力促进服务业效益、档次双提升。牢牢抓住特色旅游。完成全市旅游总体规划修编，大力发展特色文化游、农业观光游、生态湿地游和冰雪体验游，努力构建以查干湖为核心、一江两岸为风光带、特色城镇为节点的大旅游发展格局。推动天河谷温泉小镇投入运营，开工建设威尼斯水上乐园，推进华西希望“花舞松原”一期项目落地建设。牢牢抓住商贸物流。开工建设万达广场、大润发超市江北店和北京新发地现代农业综合服务园区、江苏敬亿国际商贸物流城等项目，规划建设医药物流产业园，推动上汽集团东北基地落户松原。牢牢抓住健康养生。深化与中国医药卫生事业发展基金会等机构的战略合作，加快推动癌症、糖尿病治疗中心和优生优育中心项目落地。牢牢抓住信息服务。促进互联网与传统行业深度融合，突出抓好阳光e购电子商务项目，启动实施“爱松原”工程，加快实现“全行业、全过程、全领域”信息化。深化与中国人民银行、中国联通公司、国家农业部和上汽集团等方面的战略合作，积极推进量子信息技术东北产业基地建设，加快推动国家级金融大数据中心、农业大数据示范工程落地。牢牢抓住金融保险。全市银行业金融机构本外币各项存款余额增长10%、各项贷款余额增长12%。积极支持绿色食品交易中心等要素市场建设，扎实推进设立光大银行松原分行，不断深化与国家政策性银行、国家商业银行、股份制银行的战略合作，争取保险资金投资松原。牢牢抓住房地产开发。突出规划引领，完善供地机制，合理调控节奏，统筹开发中心城区土地资源。实施“一拆三改”行动计划，加快房屋、土地征拆和招投标步伐，积极促进商业地产、温泉地产、旅游地产和养老地产发展，开工建设恒大御景湾二期、奥林匹克花园四期等项目。全面落实国家鼓励住房消费政策，有效激活房地产市场。牢牢抓住文化创意。规划建设文化创意产业园，积极开展非物质文化遗产项目展演展示活动，扶持守望乡村、启众文化传播、新视觉传媒等公司和辽金文化城发展壮大，让优势文化资源转化为文化产业和经济效益。同时，积极发展连锁经营、休闲娱乐、体育消费等新型业态，推动服务业向高端迈进。

　　（五）统筹建设美丽城乡。以优化布局、完善功能、提升品位为核心，加快把松原打造成为长春的西花园、哈尔滨的南花园、京津冀东北旅游度假基地和长三角东北避暑度假基地，让全市人民生活得更方便、更舒心、更美好。狠抓一江两岸综合开发。编制滨江生态控制性概念规划，积极争取国家第二批海绵城市试点，扎实推进松花江流域综合治理和圣湖补水工程前期工作，加快学府街和文化路等地下综合管廊建设进程，谋划建设过江隧道和城市轻轨。狠抓城市综合承载能力。加快推进查干湖机场、松原客运交通枢纽二期、天河大桥、长白铁路扩能改造和五右高速松原至扶余段建设，积极推动长春至西巴彦花铁路长岭段和国道203线肇源至善友段、望宝桥至服先段开工建设。推动宁江客运站投入使用。实施中山大街、环保大街、溪浪河大街、和平路等街路新建和改造。推进五环大街、伯都讷大街、前程西路等主街路亮化绿化美化升级，新增绿地16万平方米。加快推进“气化松原”进程，铺设天然气管网10公里，新增管道用户1万户。新建二次供水站2座，铺设取水管网10公里。启动江南污水处理厂迁移改造和江北污水处理厂二期工程，完成污泥干化工程。改造城区老旧供电设施和12条背街巷路。继续推进市规划区震害预测项目。谋划启动城区殡仪馆异地新建工程。狠抓城市精细化管理。加快“智慧城市”建设步伐，完成基础地理信息数据库、地理信息公共平台等项目建设。深入推进城市管理综合行政执法改革省级试点工作，构建权责明晰、服务为先、管理优化、执法规范、安全有序的城市管理体制。加强城区物业综合管理，开展道路交通和城市垃圾综合整治，保持城市环境整洁舒适常态化，城区机械化清扫率达到80%以上。积极开展争做“文明社区”活动，从根本上强化城市管理。狠抓美丽乡村建设。稳步推进扶余、长岭、乾安县城基础设施建设，启动建设10个特色示范镇，推动水气热供应和垃圾处理向中心镇、中心村配套延伸，建设农村公路150公里。扎实实施“厕所革命”，推广使用三零无污染厕所，加快改善农村人居环境。扎实推进环城绿化带、“三北”防护林和农田防护林建设，加快湿地恢复和“三化”草原治理，开工建设百里生态绿廊。

　　（六）扎实办好民生实事。大力实施民生行动计划，着力办好民生实事，努力打造幸福松原升级版。让百姓享有更多的就业机会。新增城镇就业4.5万人、再就业1.4万人，转移农村劳动力50万人，培训农村劳动力1.5万人，发放创业担保贷款6000万元。让百姓获得更好的社会保障。进一步提高城乡低保补助标准。继续扩大工伤、生育、失业保险覆盖面。开展政府购买城乡居家养老服务工程，探索以医养老模式。让贫困群体获得精准帮扶。启动实施脱贫攻坚战，科学制定减贫计划，强化贫困乡村基础设施建设，加快培育发展多元富民产业，确保4.8万人实现脱贫。积极为困难群体无偿提供法律援助。全面落实残疾人两项补贴制度。资助初中毕业贫困女生接受幼师和护师等职业教育。培训800名城乡贫困妇女掌握创业就业技能。让百姓享有良好的居住条件。改造棚户区60万平方米，建设、回购安置住房7500套，分配廉租房和公租房3560套，综合整治老旧小区20万平方米，改造市区危房10万平方米和农村危房3760户。让百姓得到优质的教育服务。加大教育投入，促进教育均衡发展，全面做好迎接县域义务教育国检工作。改扩建8所幼儿园及中小学校舍，新建“儿童之家”7所，筹建宁江区九年一贯制学校。让百姓享有良好的医疗服务。探索建立科学的分级诊疗体系。加快推进市中心医院异地新建项目，开工建设市妇幼保健院综合楼和市医疗紧急救援指挥中心。实施贫困妇女“两癌”筛查救助。加强甲状腺结节、病毒性肝炎等地方病、传染病的预防控制，编制发放《松原市居民健康手册》。让百姓获得丰富的文体资源。全面完成市体育馆建设，推动中国航空飞行营地项目落户松原，开工建设市冰上运动中心，完成市城区25处健身场地体育器材配建，谋划建设市水上运动中心、市青少年宫、松原大剧院和石油博物馆，实现农村文化小广场全覆盖，实施中央广播电视无线数字化覆盖工程，评选市歌、市花、市树。让百姓吃到安全的食品。建成市食品药品检验中心并投入使用，启动建设国家级绿色食品检验中心，开展食品安全快速检测3.6万批次、农产品农残检测2000批次、肉蛋奶及瘦肉精检测4660批次，确保群众饮食安全。让百姓喝上干净的水。全程监测哈达山水库等饮用水源，扎实推进江北净水厂取水和农村饮水安全工程，保障城乡居民用水绝对安全。让百姓呼吸洁净的空气。积极推进集中供热锅炉脱硫脱硝改造、棚户区及部分供热站燃煤能源替代工作，及时清理黄标车和老旧机动车，加大露天秸秆焚烧治理管控力度，加快环境保护信息化建设，抽样监测江南、江北城区有代表性的公共场所及居民室内环境质量，为实现两年后城市优良天气天数达到340天以上创造良好条件。

　　（七）锐意推进改革创新。坚持市场化改革方向，进一步发现市场、培育市场、激活市场，破除制约发展的体制机制障碍。深化国有企业改革。整合相关国有资源，组建松原城市发展投资控股集团、松原现代农业投资控股集团、查干湖生态文化旅游投资集团。实施国有林场改革。完善国有企业、国有资产、国有资本监管体制机制。深化农业农村改革。扎实推进三江口农村综合改革示范区建设。开展宅基地、水面、草场等资源确权工作，抓好农村土地承包经营权确权登记颁证工作，加快构建农村土地经营权流转市场体系，强化畜牧业监管机制，启动供销合作社综合改革。深化金融领域改革。争取设立城市基础设施、产业投资、产业并购等基金，推动成立农业金融资产管理、债券信用增进、消费保险等金融企业。支持阳光村镇银行创新发展。扎实推进宁江农信社改制，尽快组建市农村商业银行，加快扶余、乾安、长岭农信社改制步伐。积极支持和元生物、百年汉克等企业上市。做好国家农村金融综合改革试验承接工作。深化民营经济改革。围绕“大众创业、万众创新”，打破“玻璃门”、“弹簧门”和“旋转门”等隐性障碍，设立政府扶持引导基金，为民营经济发展松绑助力，培训小老板500人，新增私营企业和个体工商户6800户。深化行政管理改革。清理行政许可和行政处罚职权，落实权力清单、责任清单和负面清单制度。推行并联审批和网络审批，所有行政审批事项均进驻服务中心办理，实行一站式服务、一条龙管理，把该下放的审批权都下放到底、下放到位。强化简政放权后的事中事后监管。扎实推进机关事业单位养老保险制度改革。完成公务用车制度改革。

　　（八）积极扩大对外开放。统筹对外开放与对内合作，充分利用两个市场、两种资源，在更宽领域集聚发展要素。搭建平台促开放。统筹市县两级资源，组建松原市招商服务公司，探索在北京、上海、深圳、香港等地区设立招商服务公司分支机构，支持各县（市区）、开发区设立招商服务分公司。用活用好驻京、驻长机构和商会等载体，瞄准行业前沿技术，广泛收集领军企业投资意向，为引进战略合作伙伴提供信息保障。构建机制促开放。建立全民参与、立功奖励和县（市区）、开发区主要领导招商推进机制，设立政府引导基金，着力吸引域外重大优质项目落户松原。建立招商考核机制，各县（市区）、开发区必保分别引进开工2个、洽谈签约2个投资3亿元以上或纳税不低于1000万元的大项目，集中开展项目建设对促进经济增长、财税增收、就业增加、集约用地、改善生态环境的综合评估。建设通道促开放。加快公、铁、水、空等交通基础设施建设，构建3小时、2小时和1小时经济圈，致力于打造中蒙俄经济走廊开发开放新门户、面向东北亚和俄罗斯地区跨境贸易重要集散地，深化与美国、以色列、泰国、台湾等国家和地区的战略合作，积极融入长吉图开发开放先导区和中韩自贸区，广泛与中华两岸连锁经营协会和全国招商引资网、省投资促进网等平台开展合作，开创对外开放新格局。宣传推介促开放。谋划与国内外大企业、大财团联合举办国际生物质产业、绿色农业高端论坛。启动“查干湖+”行动计划，办好查干湖冬捕节、荷花节，筹办查干湖国际桥牌邀请赛、少数民族歌会、电子竞技大赛和摄影大赛。通过加大宣传力度，促进对外开放取得丰硕成果。

　　（九）着力打造平安松原。适应社会发展新趋势，不断提高社会治理能力和社会治理水平。全面加强社会治理。启动“七五”普法工作，提升全民法治意识。完成村民委员会第十次换届选举。依法加强宗教管理。鼓励支持社会组织参与社会治理和公共服务。实施和谐社区建设工程。切实保障安全生产。全面落实安全生产主体责任，建立健全事故约谈、企业黑名单等制度，扎实开展安全生产专项整治，实现“五级五覆盖”，坚决遏制重特大事故发生。着力维护社会稳定。加强社会矛盾化解，落实属地稳控和领导包保责任，强化信访法律服务中心职能，提高信访工作法治化水平。依法打击违法犯罪行为，加强立体化社会治安防控体系和市应急联动指挥平台、“天网工程”建设。进一步提高预防和处置地震等突发公共事件能力，切实保障人民生命财产安全。

　　（十）全面加强政府建设。坚持依法行政。积极主动接受市人大及其常委会法律监督、工作监督和市政协民主监督，切实提高人大代表建议和政协提案办理实效。加快推进重点领域行政立法工作。落实公众参与、专家论证、风险评估、合法性审查、集体讨论决定等决策机制，进一步提升政府决策的民主化、科学化水平，全面增强政府工作的透明度和公信力。坚持为民执政。始终把增进民生福祉作为一切工作的出发点和落脚点，时刻把群众的安危冷暖放在心上、扛在肩上、抓在手上，主动倾听群众呼声，真切关心群众疾苦，及时解决群众诉求，坚持不懈办好民生实事，努力让发展成果更多地惠及人民群众。坚持高效理政。坚持一张蓝图绘到底、一张表格考到底，一以贯之抓落实。深入开展机关作风建设，强化政府绩效管理工作，精简各类会议和文件，切实改进文风会风，把主要精力用在办实事、抓落实上。坚持敢于担当、善于担当、体现担当，坚决做到危险面前不退让、困难面前不退缩、机遇面前不犹豫、责任面前不推托。倡导马上就办、雷厉风行的工作作风，以高效率赢得高效能，以快节奏推进快发展。坚持从严施政。严厉整治不作为、乱作为和“吃拿卡要”等突出问题，大力优化经济发展软环境，坚决纠正损害群众和客商利益的不正之风。深入推进廉政建设和反腐败工作，全面落实“两个主体责任”，认真践行“三严三实”，严肃查处各类违纪违法案件，切实抓好廉政建设“起步一公里”、廉政落实“最后一公里”，团结干事，共同干净，坚决维护风清气正的政治局面。

　　各位代表！2016年作为“十三五”开局之年，开启了我市振兴发展的新征程。站在新的起点上，我们深知使命光荣、任重道远。让我们在市委的坚强领导下，紧紧依靠和团结带领全市各族人民，振奋精神、团结一心，攻坚克难、埋头苦干，锐意进取、开拓创新，为全面实现“十三五”规划宏伟蓝图、谱写全面建成小康社会新篇章而努力奋斗！
